# Maternal Education, Fertility, and Child Survival in Comoros

**DOI:** 10.3390/ijerph15122814

**Published:** 2018-12-10

**Authors:** Abayomi Samuel Oyekale, Thonaeng Charity Maselwa

**Affiliations:** Department of Agricultural Economics and Extension, North-West University Mafikeng Campus, Mmabatho 2735, South Africa; thonaengmaselwa@gmail.com

**Keywords:** child survival, education, fertility, Comoros

## Abstract

Reduction in child mortality is a demographic progress of significant socioeconomic development relevance in Africa. This paper analyzed the effect of maternal education and fertility on child survival in the Islands of Comoros. The 2012 Demographic and Health Survey (DHS) data were used. A two-stage probit regression method was used for data analysis. The results showed that about 75% of the children’s mothers had given birth to between one and five children, while more than half did not have any form of formal education. The results of the two-stage probit regression showed that while child survival reduced significantly (*p* < 0.05) with the age of the heads of households, residence in the Ngazidja region, being born as twins, mother’s number of business trips, and number of marital unions, it increased with maternal education, fertility, male household headship, and the child being breastfed immediately after birth. It was concluded that efforts to enhance maternal education would reduce child mortality. It is also critical to promote child breastfeeding among women, while regional characteristics promoting differences in child mortality in Comoros Islands should be properly addressed with keen focus on the Ngazidja region.

## 1. Introduction

The rate of mortality among children under five is one of the internationally acceptable indicators for assessing a country’s progress towards promotion of sustainable healthcare service delivery and economic development [1]. These indicators also clearly highlighted part of the Sustainable Development Goals (SDGs), which present some international renewed commitments towards the global fight against child mortality. Specifically, the child mortality-related SDG seeks to end “preventable deaths of newborns and children under the age of 5 years, with all countries aiming at reducing neonatal mortality to at least as low as 12 deaths per 1000 live births and under five mortality to at least as low as 25 deaths per 1000 live births” [2].

In 2015, infant and under-five mortality rates in Comoros were estimated at 67.2 per 1000 live births and 92 per 1000, respectively [3]. Although these demographic profiles are slightly better than those of some African countries [4], they are still significantly higher than what is obtainable in many developed countries. Being one of the smallest countries in Africa [3,5], these statistics also provide some indications that after many years of political instability, the country is being repositioned for medium-term economic growth and development. This can be proved with consistency in Gross Domestic Product (GDP) growth rates, which for the first time in many years exceeded the rate of population growth rate in 2011 [6].

The Comoros’ 2005 national health policy itemized some programs for achieving some of the Millennium Development Goals (MDGs). This policy seeks to have some positive impacts on some demographic indicators, such as child mortality, maternal fertility and mortality. The Comoros’ 2013 MDG report also attested to the country’s drive towards meeting some targets in terms of gender equity in universal primary education (UPE) and reduction in maternal and child mortality [6]. Low literacy rate among women—49.3% in 2003 as compared to 63.6% for men [5]—is a reflection of formal education’s inequality. Efforts are now in place to promote girl child education [7,8] and remove some spatial barriers to healthcare services [9]. Maternal fertility is another major issue of concern in Comoros. Available data indicate that fertility was higher in rural areas (6.1), when compared with urban areas (5.4) [4].

Maternal education and fertility significantly influence a child’s health and survival remains an important area of research inquiry today. Theoretically, the frameworks for explaining the relationships between maternal education and child mortality are well documented in economic and public health literature. Cadwell’s hypothesis emphasizes the role of women’s education in promoting child’s health [10]. This had been reformulated through several analytical frameworks developed by Meegama [11], Garenne and Vimard [12], and Mosley and Chen [13]. Based on these frameworks, the association between maternal education and child’s health had been confirmed [14,15,16,17,18,19]. Education is bound to improve efficiency of producing health investments through proper utilization of health-related knowledge [20]. Therefore, individuals’ marginal productivity of health inputs would increase with their years of education.

Some previous studies have found positive correlation between maternal education and child’s health status, while several other studies have confirmed negative association between child mortality and maternal education [14,15,16,17,18,19]. However, the impact of maternal education on child mortality is often assumed to be inflated due to inability to control for other unobservable maternal characteristics such as inherent ability [1]. It had been noted that such indicator of ability like maternal intelligence quotient could constitute a significant determinant of child mortality, even in absence of any formal education [21,22]. 

In addition, reduction in maternal fertility is often facilitated by educational attainments [23], thereby contributing to overall child’s health and development [24]. The notion of time constraints that educated mothers often face due to a high probability of participating in the labor market emphasizes their generally low fertility rate [25,26]. This could also imply a better health outcome for their children, due to the consistent flow of income required to meet some basic needs of the household’s members. Another line of reasoning is the indicative association that generally exists between educated women and educated men [21,23,24]. With or without some cultural and religious influences, educated women are often expected to be married to educated men, who would have some stable sources of income for meeting the needs of their households [27,28,29]. This analytical paradigm concludes the influence of education in familial engagements and the role it plays in securing sustainable future for the children belonging to those households.

In some previous studies, policy decisions on implementation of compulsory primary education in low income countries had been linked to several research findings. Dincer et al. [30] found that maternal education influenced fertility and use of contraceptives in Turkey. Gunes [31] reported the influence of maternal education on raising healthy children and generally low incidence of children born with low birth weights. In addition, Osili and Long [25] found that in Nigeria, maternal fertility reduced as a result of introduction of universal primary education. Cleland and Ginneken [32] found that increasing maternal education by one year would reduce under-five children’s mortality rates by 7–9% in some low-income countries. In Bangladesh, it had been reported that mortality among under-five children was 45% lower for those kids whose mothers attained secondary education when compared to those with illiterate mothers [33]. Akter et al. [1] found that in Bangladesh, attainment of secondary education by mothers contributed to reduction in the odds of under five mortality by 38%. Using data for 175 countries, Gakidou et al. [34] estimated that maternal education accounted for 51.2% of the 8.2 million reduction in under-five mortality between 1970 and 2009.

Moreover, understanding the role of maternal education and fertility in explaining child survival in low income countries like Comoros is of essential policy relevance. Although Lachaud [35] analyzed the determinants of child mortality in Comoros, no consideration was given to maternal fertility as a variable or the likelihood of maternal education being endogenous within the specified model. This purpose of this paper is to bridge existing gaps in academic literature by analyzing the effects of maternal education and fertility on child survival in Comoros. This was implemented through some econometric testing for ascertaining our suspicion on likely endogeneity of maternal education and fertility in the child survival model. These econometric procedures were followed in order to ensure that estimated parameters in our specified models are unbiased and consistent.

## 2. Methods

### 2.1. The Study Area

Comoros comprises of three Islands—Anjouan (Nzwani), Grande Comore (Ngazidja) and Moheli (Mwali)—which are offshoots of volcanic eruptions in the Indian Ocean. However, the fourth island, which is known as Mayotte Island, is administratively controlled by France [36]. About 75% of the population in Comoros lives in rural areas, while about half of the population resides in Grande Comore Island [37]. With a surface area of about 1660 km^2^ and an estimated population of approximately 790,000 people in 2015 [38], Comoros is a small country with splendid natural resources and biodiversity. The country is endowed with agro-biodiversity resources, which reemphasizes the relevance of the agricultural sector that often accounts for about 40% of the gross domestic products (GDP) [39]. Poverty is one of the major development challenges in Comoros, especially in rural areas. In 2004, 45% of the households were poor [40], although the country’s Human Development Indices (HDI) has increased from 0.451 in 2005 to 0.497 in 2015 [41].

### 2.2. The Data and Sampling Methods

The 2012 Demographic and Health Survey (DHS) data for Comoros were used for this study. The sampling proceeded with selection of 252 clusters, from where 5041 households were randomly selected. Of these households, 4482 were successfully sampled from the 4656 that were occupied. The response rate for the entire survey was 96% in the rural and urban areas. Furthermore, 5719 women who were between 15 and 49 years of age were eligible to participate in the women’s questionnaire. Out of these women, 5329 were successfully interviewed. The data were collected with structured questionnaires that were administered by trained enumerators. The questionnaires were also pretested in Moroni City and some nearby rural areas. The final questionnaire for the survey benefitted from some observations that were made during the pretesting processes [42]. This study used the SPSS file format of the data file that was tagged KMBR61FL. This file comprises of the list of all the children that were ever born by those eligible women, whether deceased or alive. The file comprises of 11,479 listed children from 2934 women. In the analyses that were carried out in this study, none of the respondents was dropped due to completeness of data points for those selected variables.

## 3. Analytical Models

### Two Stage Probit Regression

Analyzing the impact of maternal education and fertility on child survival requires some robust econometric procedures. The first issue is to verify whether maternal education and fertility are truly endogenous. This was implemented with Durbin–Wu–Hausman (DWH) test [43,44,45] that was implemented within the standard probit regression. We begin the modeling processes by specifying the child survival model as follows:

(1) $$Y_{i}=\alpha +\beta_{m}\overset{23}{\underset{m=1}{\sum }}X_{mi}+\gamma F_{i}+\rho Ed_{i}+e_{i}$$

The dependent variable, $Y_{i}$ is dichotomous in nature. This is the child survival variable which was coded 1 if the child was alive at the time of the survey and 0 otherwise. Similarly, $\alpha ,\;\beta_{m}$, γ and ρ are the estimated parameters. The independent variables, $X_{mi}$ are Ndzouani region (yes = 1, 0 otherwise), Ngazidja region (yes = 1, 0 otherwise), children are twins (yes = 1, 0 otherwise), gender of child (male = 1, 0 otherwise), urban residence (yes = 1, 0 otherwise), Muslim religion (yes = 1, 0 otherwise), male household headship (yes = 1, 0 otherwise), age of household head (years), telephone access (yes = 1, 0 otherwise), share toilet (yes = 1, 0 otherwise), number of business trips in the past one year, health insurance access (yes = 1, 0 otherwise), away for more than one month (yes = 1, 0 otherwise), mosquito nets ownership (yes = 1, 0 otherwise), not smoking tobacco (yes = 1, 0 otherwise), number of unions, child breastfed immediately (yes = 1, 0 otherwise), mothers’ age at first birth, wealth index, listening to radio (yes = 1, 0 otherwise), reading newspapers (yes = 1, 0 otherwise) and watching TV (yes = 1, 0 otherwise). $F_{i}$ is maternal fertility (number of children born whether dead or alive) and *Ed_i_* is the mothers’ years of education. Endogeneity of maternal education and fertility were confirmed with DWH test. The assumed specifications of the endogenous regressors are presented in Equations (2) and (3).

(2) $$F_{i}=\theta +\mu_{m}\overset{18}{\underset{m=1}{\sum }}X_{mi}+\pi_{k}\overset{3}{\underset{k=1}{\sum }}Z_{ki}+\vartheta Ed_{i}+u_{i}$$

(3) $$Ed_{i}=\omega +\varphi_{m}\overset{18}{\underset{m=1}{\sum }}X_{mi}+\varepsilon_{k}\overset{3}{\underset{k=1}{\sum }}Z_{ki}+\tau F_{i}+v_{i}$$

In order to estimate Equations (2) and (3), some instrumental variables ($Z_{ki}$) were identified. These variables are expected to be highly correlated with the endogenous regressors (education and fertility), but not correlated with child survival. Although different instruments had been used for similar studies, selection of instruments needs proper cautions in order to avoid weak instruments. In this study, we selected child’s birth order and gender of the first child (coded as male = 1 and 0 otherwise) as instruments for maternal fertility. The order of birth is directly related to maternal fertility. Also, the traditional notion of high preference that is often given to male children can influence maternal fertility. Therefore, the search for a male child can promote high fertility. Moreover, years of education of the husband was selected to instrument maternal education because in some instances, educated men do have preference to marry educated women [21,23,24]. The selected endogenous variables must not show high correlation with the dependent variable in Equation (1). Table 1 shows the correlation matrix between the selected instruments and the endogenous variables across the different levels of econometric analyses. In all cases, child survival shows low correlation with these instrumental variables.

The DWH test requires estimation of Equations (2) and (3) separately and computation of the values of their residuals. These residuals are then jointly introduced into Equation (4) for a standard probit regression.

(4) $$Y_{i}=\chi +\lambda_{m}\overset{18}{\underset{m=1}{\sum }}X_{mi}+\delta F_{i}+\zeta Ed_{i}+\epsilon v_{i}+\eta u_{i}+z_{i}$$

Technically, Equation (4) is the two-stage probit regression. If ϵ and η are statistically significant (*p* < 0.05), maternal education and fertility are endogenous. In this case, ignoring their endogeneity property as presented in Equation (1) would produce biased and inconsistent estimators. However, should their parameters show statistical insignificance (*p* > 0.05), Equation (1) should be ideally estimated with a standard probit regression.

## 4. Results

### 4.1. Descriptive Statistics of Children’s Demographic Characteristics

Table 2 shows the descriptive characteristics of the listed children in the lower segment and those of their mothers in the upper segment. The results show that while 57.2% of the children’s mothers were from urban areas in Mohéli region, 68.3% and 59.9% were from rural areas in the Ndzouani and Ngazidja regions, respectively. In the full dataset, only 40.3% of the children’s mothers were from urban areas. The table also shows educational attainments of the children’s mothers. It reveals that for the children from Ngazidja region, about one out of four of their mothers had secondary education, while only 4.4% had higher education. In Mohéli and Ndzouani regions, 55.2% and 70.0% of the children’s mothers respectively had no formal education. In the full dataset, 54.8% and 3.6% of the children’s mothers had no formal education and had higher education, respectively. In addition, about 75.7% of all the children’s mothers had given birth to between 1 and 5 children, while 89.7% and 90.7% did not record death of boy and girl child, respectively. Also, 51.4% of all the children were males, 2.3% were born as twins and 21.2% were breastfed immediately.

### 4.2. Determinants of Maternal Education and Fertility

Table 3 are the estimated parameters using OLS regression for the determinants of maternal years of education and fertility based on the specification in Equations (2) and (3). These results were generated for the full dataset, those that were under-five and those that were 0–12 months old. The models all show statistical significance (*p* < 0.01). In addition, the results indicated that the selected instrumental variables show statistical significance (*p* < 0.01) for the two endogenous variables. Essentially, these results were utilized to generate the residuals, which are to be integrated into Equation (4) in order to confirm if maternal education and fertility are truly endogenous in Equation (1).

### 4.3. Demographic, Maternal and Child Characteristics Influencing Child Survival

The results in Table 4 are the two-stage probit regression estimates of Equation (4). These results were generated for the full dataset, under-five children and 0–12 months-old children. In all the results, the F-statistics show that the models are statistically significant (*p* < 0.01), and the residual parameters are all with negative sign and statistically significant (*p* < 0.01) except that of fertility in the models for all children and those under-five, which are statistically insignificant. At least, the results in the infant model provide some indications that both maternal education and fertility should be treated as endogenous regressors. Therefore, estimating Equation (1) with the standard probit regression would produce biased and inconsistent estimators.

The results in Table 4 are for the two-stage probit regression, which were carried out for the full dataset, children 0–59 months (under-five) and children 0–12 months old (infants). There was no serious problem of multicollinearity among the explanatory variables based on the very low values of the computed variance inflation factor (VIF) [46]. Specifically, the maximum computed VIF of 1.27 implies that the average coefficient of determination among the explanatory variables was approximately 0.21. The computed Wald Chi Square values for each of the models show statistical significance (*p* < 0.01).

The results also show that the parameter of mothers’ years of education are of note in all the estimated models and show statistical significance (*p* < 0.05) in the under-five and infant models. These results imply that education attainment significantly increased the surviving probabilities of children under-five and infants. Maternal fertility similarly had positive and statistically significant (*p* < 0.01) parameters in the models for the under-five and infants. These imply that maternal fertility increased the probabilities of under-five and infant survival. The parameters of Ngazidja regional variable show statistical significance for all the models (*p* < 0.05). These results show that when compared with those from Mohéli region, all the children in Ngazidja region had a significantly lower probability of surviving. However, in the infant (under-five models), the probability of child survival significantly increased (decreased) respectively by residing in Ngazidja region.

The results in Table 4 further show that those children born as twins had significantly lower probability (*p* < 0.01) of surviving, irrespective of their age. Also, being a male significantly increased the probability of surviving among infant children. The parameter of urban residence in the model for “all children” shows statistical significance (*p* < 0.01). This shows that children whose mothers resided in urban areas had significantly higher probability of surviving, when compared with their counterparts from rural areas. However, in the infant model, the parameter of urban residence is with negative sign but statistically insignificant. The parameter of religious affiliation shows statistical significance in the full dataset and infant models (*p* < 0.05). These imply that irrespective of their age, children of Muslim mothers had higher probability of surviving when compared with those practicing other religions. However, survival of infants significantly reduced among Muslim mothers.

Also, the parameters of households’ head gender show statistical significance (*p* < 0.05) in the three estimated models. These results imply that compared to female headed households, children from male-headed households had higher probability of surviving. The parameters of age of household heads in the models for children under-five and 0–12 months show statistical significance (*p* < 0.01). These results imply that survival probability among under-five children and those 0–12 months old decreased as the age of household heads increased. Also, the number of business trips parameters show statistical significance (*p* < 0.05) in the model for all children. These results show that as the number of mothers’ business trips increased, the probability of surviving decreased. The parameters for the number of unions variable are with negative sign and statistically significant (*p* < 0.01) for all the models. These results show that the probability of child survival decreased as the number of unions increased. In all the estimated models, being breastfed immediately after birth significantly (*p* < 0.01) increased the probability of child survival. In the infant model, the parameters of access to telephone, access to health insurance and ownership of mosquito nets are all statistically significant (*p* < 0.05) with positive sign. These results show that infant survival significantly increased among households with access to telephone, health insurance and mosquito nets. In the under-five and infant models, mothers’ age at first birth parameters are with positive sign and statistically significant (*p* < 0.01). These results show that as the mothers’ age at first birth increased, probabilities of under-five and infant child survival increased. Among the variables that were used to capture access to information, the parameters of listening to radio, reading newspapers, and watching television are statistically significant in the infant model. These results indicate that probabilities of infant survival decreased with listening to radio and reading newspapers, while it increased with watching television.

## 5. Discussions

The results show some regional difference in child survival. Specifically, using Mohéli as the baseline variable, residence in Ngazidja region reduced the probability of child survival in the full model, but increased infant survival. Previously, the World Health Organization [47] reported that Ngazidja region had the highest mortality among infant and under-five children. United Nations International Children’s Emergency Funds (UNICEF) [48] submitted that among the major causes of child mortality in Comoros are poor access to safe drinking water, lack of hygienic practices and improved sanitation, sexual violence that is often promoted by persistent poverty and weaknesses of existing institutional capacities to address reported cases.

The results also indicated that ownership of mosquito nets increased survival among infant children. Therefore, another vital issue that may affect child’s health in Comoros is the regional pattern of distribution and severity of some diseases. The foremost example is malaria, which is known to account for high mortality among children in sub-Saharan Africa and ranked second as a major cause of premature death among Comorians between 1990 and 2010 [49]. There are some fundamental differences in the biological characteristics of malaria parasites from Great Comoro (Njazidja) and those from the other highlands. More specifically, malaria parasites in the Great Comoros have higher genetic mutation frequency that is well associated with some cases of resistance [50,51]. More importantly, Chakir et al. [52] reported that there have been some interventions to control malaria in entire the Moheli region since the end of 2007 based on distribution of ACT anti-malaria drug. Also, some initiatives had been taken to replicate the program in Ndzouani region. Currently, the efficacy of the initiative is being tentatively confirmed with more than 98% decrease in malaria incidence in the Mohéliand Ndzouani regions.

Urbanization is one of the major factors influencing child survival. The results from the full dataset model show that the probability of child survival increased among urban households. Similar findings had been reported in some previous studies. For instance, Antoine and Diouf [53] in a study on Ghana, Senegal, Kenya, Benin and Cameroon and Sastry [54] on Brazil highlighted that due to disparities between urban and rural areas in terms of social infrastructure like access to immunization services, portable water, improved sanitation and healthcare facilities, children born in urban areas seem to have higher likelihood of surviving. Residence in urban area can as well influence child survival through the notion of relatively higher income and educational attainments of urban people [55]. In some instances, maternal fertility in urban areas could be 30% lower than what is found in rural areas [56,57].

In this study, the parameter of maternal fertility is with negative sign and statistically insignificant in the full dataset model. However, in the under-five and infant models, fertility increased the probability of child survival. Theoretically, there are many routes through which maternal fertility can influence child survival. Becker and Lewis [58] and Becker and Tomes [59] indicated that parents’ decisions on the desired number of children and individual child investments are jointly taken. This underscores the fact that based on the quantity–quality model, the notion of perpetual decline in the level of resources that are available to promote child’s well-being as family size increases cannot be underestimated. In addition, from the medical point of view, the hygiene hypothesis notes that child’s exposure to disease causing pathogens increases as family size increases [60]. Such exposure can culminate into some illnesses, which if not well treated can reduce the probability of child survival. There have been some studies that addressed endogeneity of maternal fertility in explaining child’s health and mortality. It had been shown that large family size reduces access to clean water and improved sanitation [61]. It can also reduce child’s nutritional status [62] and their heights [63], which consequently result in poor health status [64].

The other routes through which maternal fertility can facilitate child survival should be considered. The first argument is that preference for large family size can be independently taken irrespective of who survives or dies among the children. Comoros, being an Arab nation, presents some peculiarities given its constitutional allowance to polygamy and divorce. However, where maternal fertility is driven by previous child mortality, child survival can be enhanced as fertility increases due to already acquired childcare experiences. It had also been noted that high fertility may be induced by child mortality insurance decision. This is a situation whereby mothers oblige to giving birth to many children in anticipation that mortality might be inevitable. This presupposes that children born to highly fertile women can still have higher probability of survival. In some previous studies, Gubhaju [65] submitted that high mortality may occur among children in the first order because they may have been born to inexperienced young mothers. However, using Kenyan data, Kabubo-Mariara et al. [66] found positive correlation between child mortality and their birth order. Another important consideration is that in some cultural settings, there is high preference for certain gender. This often induces high maternal fertility as couples keep searching for their certain gender, irrespective of survival status of other children [67].

Mother’s year of education is also one of the determinants of child survival. In the results, years of education increased the chances of child survival in the under-five and infant models. The impact of maternal education on child survival had been evaluated through its influence on child’s health status [1]. In some previous studies, maternal education had been emphasized as a critical determinant of child survival in Bangladesh [17], Uganda [16,19], Zimbabwe [17], Malawi [18], and Ethiopia [68].

The results further indicated that children that were born as twins had lower probability of surviving across the different estimated models, when compared with single births. Similar results had been reported by Schoeps et al. [69] and Lartey et al. [70]. In the context of socio-economic conditions in Comoros, inadequate nutrition, inadequate healthcare facilities and persistent poverty can reduce survival of twin children. It had also been noted that inability to secure mother’s attention can reduce probability of child survival. This notably increases when the mother has twins. In a recent study, Monden and Smits [71] analyzed several DHS data for many African countries and concluded that mortality among twins was higher. Mortality among twins can be very high due to their extreme vulnerability, which results from high risk of complications for both the mothers and the children, and higher likelihood of being born with very low birth weights. In some other instances, immediate care by mothers to twin children may be delayed due to some complications accompany their birth [71,72,73]. Some other studies from Bangladesh [74], Nigeria [75], Ghana [70], and some countries in southern and eastern Africa [76] had reported similar findings.

The number of business trips that were engaged by the mothers in the past year reduced child survival in all the estimated models. These results are in line with empirical expectations, given that availability of mothers at home enhances the quality of childcare, which increases child survival [77]. Male household headship also increased child survival across the different models that were estimated. There have been mixed results on the role of household heads’ gender in child’s survival. In some previous studies, it had been argued that male headship comes with many financial benefits that can promote healthy living among children, and reduce their susceptibility to sicknesses and diseases [78,79]. In some other studies, however, female headship enhanced child survival [80,81] on the account that the mothers are able to seek timely healthcare services when headed by women. In addition, children that were born to households that were headed by old people had lower probability of survival. This is in line with the findings of Izugbara [82] using Nigerian data.

Initiation of timely breastfeeding for children increased child survival across all the levels of analyses. This finding reemphasizes the importance of child’s breastfeeding for enhancing their health and survival. Breast milk is highly nutritious and the World Health Organization recommends exclusive breastfeeding for six months [83]. In a study by Fosu-Brefo and Arthur [83], the importance of timely breastfeeding of children for promoting the health and survival of Ghanaian children was emphasized. However, some other studies had highlighted the role of breastfeeding in enhancing child survival [84,85].

The number of marital unions by child’s mother reduced child survival. This is a pointer to the role of marital stability in promoting the health of children [86,87]. In some previous studies, Carr and Springer [88] and Waite and Gallagher [89] analyzed the effect of marital status on child’s health. Several other studies found that children that were born to married couples had higher probability of surviving than those born to divorced homes [90,91]. Gurmu and Etana [91] specifically found significant positive relationship between probability of stunting and single-parent nuclear family in Ethiopia. Using the datasets from Cameroon, Democratic Republic of Congo and Nigeria, Ntoimo, and Odimegwu [90] found that under-five mortality risk was significantly higher for children born to single mothers.

Among under-five children, being a male child significantly reduced the probability of survival. Some previous studies have found lower chances of survival for male children [92]. Specifically, Pongous [92] argued that infant mortality is higher among male children due to gender-related differences that are associated with their genetic and biological composition. It was noted that boys are likely to be more susceptible to premature death resulting from diseases. The results also showed that the age at which mothers gave birth to the first child increased child survival. This is expected because teenage pregnancy could promote some pregnancy complications that can reduce child survival. Specifically, early pregnancy can promote anemia [93,94,95], while the children could have low birthweights [93,96,97,98]. Finally, uptake of health insurance enhances infant survival. This is an important attestation to the role that removal of barriers to healthcare service access could have on child’s health [99]. Health insurance can facilitate maternal and child’s health through access to improved medical services that are able to reduce infant mortality. Similar results had been reported for a study in Ghana [100,101].

## 6. Conclusions

This study analyzed the effects of maternal education and fertility on child survival with proper correction of their endogeneity. Based on the main results from data analyses, some conclusions could be reached as a way of fathoming a policy design process to address child mortality in Comoros. Specifically, urban development promises significant reduction in child mortality. Some of the basic advantages that urban children usually have over their rural counterparts are proximity to health care facilities and programs like nutrition enhancement and immunization. There is the need to ensure that rural areas in Comoros have fair coverage of basic social services for promoting child’s health. In addition, based on some regional differences in the distribution of child mortality across the regions in Comoros, proper reevaluation of equity and impact of basic social services in enhancing households’ health would reduce child mortality, especially with specific focus on the Njazidja region. A very good example is the need to duplicate the program on mass control of malaria program in the Island of Njazidja as well. It was also found that maternal education and age at first birth are critical in increasing child survival in Comoros. There is the need to promote girl-child education and discourage teenage pregnancy and/or marriage in Comoros. More importantly, with education, it is expected that maternal fertility can be reduced with some other associated health benefits. Moreover, specially designed programs to promote the survival of male and twin children promise to increase child survival in Comoros. There is also the need to facilitate sensitization on the negative consequences of mothers’ absenteeism and divorce on child survival. Finally, initiatives to promote timely breastfeeding of children and access to health insurance promises significant impact on increasing child survival.

## Figures and Tables

**Table 1:** Correlation Matrix of Endogenous Variable/Regressors and Selected Instrumental Variables.

	Child Survival	Education	Fertility	Child Survival	Education	Fertility	Child Survival	Education	Fertility
	All Children			Children 0–59 Months			Children 0–12 Months		
Birth Order	−0.0196	−0.2289	0.6124	0.0119	−0.3278	0.8270	0.0247	−0.2778	0.6921
Husband’s Years of Education	0.0411	0.4239	−0.2840	0.116	0.4456	−0.2972	0.1452	0.4209	−0.3139
First Child is Male	−0.0152	0.1113	−0.2258	−0.0525	0.1649	−0.3017	−0.0916	0.0962	−0.2383

**Table 2:** Descriptive statistics of selected characteristics of children in the Comoros’ region.

Demographic Characteristics	Mohéli		Ndzouani		Ngazidja		All Respondents	
Children’s Mothers	Freq	%	Freq	%	Freq	%	Freq	%
Urban	318	57.2	334	31.7	531	40.1	1183	40.3
Rural	238	42.8	721	68.3	792	59.9	1751	59.7
**Highest Year of Education**								
No education	1327	55.2	3111	70.0	1865	40.1	6303	54.8
Primary	581	24.2	618	13.9	1417	30.5	2616	22.8
Secondary	435	18.1	558	12.6	1145	24.6	2138	18.6
Higher	62	2.6	141	3.2	206	4.4	409	3.6
**Total Children Born**								
1–5	389	70.0	748	70.9	1084	81.9	2221	75.7
6–10	153	27.5	288	27.3	229	17.3	670	22.8
11–15	14	2.5	18	1.7	10	0.8	42	1.4
>15	0	0.0	1	0.1	0	0.0	1	0.0
**Sons Who Have Died**								
0	496	89.2	956	90.6	1180	89.2	2632	89.7
1	49	8.8	79	7.5	121	9.2	249	8.5
2	7	1.3	17	1.6	19	1.4	43	1.5
3	4	0.7	1	0.1	3	0.2	8	0.3
4	0	0.00	2	0.2	0	0.0	2	0.1
**Daughters Who Have Died**								
0	506	91.0	953	90.3	1202	90.9	2661	90.7
1	42	7.6	87	8.3	103	7.8	232	7.9
2	7	1.3	12	1.1	16	1.2	35	1.2
3	0	0.0	3	0.3	1	0.1	4	0.1
4	1	0.2	0	0.0	1	0.1	2	0.1
**Children**								
Breastfed immediately	383	15.9	920	20.7	1136	24.4	2439	21.2
Child is alive	2270	94.4	4198	94.5	4340	93.3	10,808	94.0
Twins	43	1.8	96	2.2	123	2.7	262	2.3
Male	1285	53.4	2261	50.9	2368	50.9	5914	51.4

**Table 3:** Determinants of Maternal Fertility and Years of Education using Ordinary Least Square (OLS) regression.

	All Children				Under 5 (Children 0–59 Months)				Infants (Children 0–12 Months)			
	Fertility		Education		Fertility		Education		Fertility		Education	
	Coeffic	Z Stat	Coeffic	Z Stat	Coeffic	Z Stat	Coeffic	Z Stat	Coeffic	Z Stat	Coeffic	Z Stat
Fertility	-	-	−0.25	−15.7	-	-	−0.26	−6.2	-	-	−0.21	−4.1
Education	−0.08	−15.7	-	-	−0.04	−6.2	-	-	−0.07	−4.1	-	-
**Region**												
Ndzouani region	0.09	1.6	−1.00	−10.8	0.11	1.5	−0.90	−4.9	0.16	1.0	−0.83	−2.6
Ngazidja region	−0.53	−9.0	−0.56	−5.5	−0.11	−1.4	−0.52	−2.6	−0.10	−0.5	−0.53	−1.5
Child is twins	1.01	8.5	0.24	1.2	1.22	8.2	0.09	0.2	1.42	4.8	0.40	0.8
Gender of child	0.04	1.1	0.05	0.8	−0.04	0.7	0.05	0.4	−0.17	−1.4	−0.27	−1.2
Urban residence	−0.03	−0.7	0.59	8.2	−0.11	−2.0	0.90	6.3	−0.17	−1.1	1.22	5.0
Muslim religion	−0.32	−1.6	0.01	0.1	0.15	0.6	−0.46	−0.7	0.41	0.9	−0.05	−0.1
Male head of household	−0.09	−2.4	−0.07	−1.0	−0.23	−4.5	−0.03	−0.3	−0.36	−2.9	−0.15	−0.7
Age of head	0.04	25.9	0.01	2.6	0.02	11.5	0.02	3.3	0.04	8,8	0.02	1.8
Telephone access	−0.15	−2.3	0.57	4.9	−0.05	−0.5	0.04	0.2	−0.17	−0.7	−0.14	−0.3
Share toilets	−0.12	−2.5	0.08	1.0	−0.10	−1.8	0.08	0.5	−0.18	−1.2	0.20	0.8
Number of business trips	0.00	−0.2	0.02	5.3	0.00	0.3	0.01	1.9	0.00	−0.2	0.01	1.4
Health insurance access	−0.10	−1.2	1.24	8.5	0.01	0.1	1.04	3.8	−0.19	−0.7	0.46	1.0
Away for more than one month	−0.09	−1.5	−0.06	−0.6	−0.20	−2.3	0.26	1.2	−0.28	−1.2	0.69	1.7
Mosquito nets ownership	−0.15	−3.3	0.22	2.8	−0.10	−1.5	0.34	2.1	−0.20	−1.3	0.15	0.6
Not smoking tobacco	−0.02	−0.5	0.41	6.4	−0.07	−1.4	0.42	3.5	−0.06	−0.5	0.59	2.7
Number of unions	0.16	5.2	−0.38	−7.3	0.14	3.6	−0.50	−5.1	0.19	2.0	−0.52	−3.1
Breastfed immediately	0.01	0.3	−0.12	1.6	−0.28	−5.3	−0.09	−0.7	−0.56	−4.3	−0.29	−1.2
Age at birth	−0.11	−27.3	0.09	12.8	−0.05	−10.4	0.08	6.1	−0.10	−7.9	0.10	4.3
Wealth index	0.00	−3.8	0.00	16.5	0.00	−1.7	0.00	9.4	0.00	−1.2	0.00	4.3
Child’s birth order	0.59	64.3	0.02	1.2	0.80	70.8	−0.04	−0.9	0.70	26.7	−0.05	−0.8
Spouse education	−0.03	−8.3	0.15	23.3	−0.02	−3.3	0.15	12.5	−0.03	−2.4	0.14	6.9
Radio	−0.06	−1.5	0.24	3.4	0.01	0.2	0.19	1.5	0.04	0.3	0.39	1.7
Newspapers	0.19	2.9	4.45	44.3	0.00	0.0	4.62	24.8	0.03	0.1	4.83	14.5
Television	−0.16	−3.6	0.17	2.3	−0.11	−1.8	0.28	1.9	−0.18	−1.3	0.16	0.7
First child is male	0.16	2.6	0.32	3.0	0.26	3.1	0.53	2.5	0.31	1.57	0.12	0.3
Constant	4.99	20.3	1.22	2.9	2.04	6.6	1.94	2.4	3.08	4.7	1.12	1.0
Number of jobs	11,497.00		11,497		3605		3605		1198		1198	
F (23, 11473)	467.85		389.65		381.38		133.74		69.73		41.86 ***	
Adj R-squared	0.51		0.47		0.73		0.49		0.60		0.47	
Mean VIF	1.37		1.39		1.40		1.54		1.44		1.49	

Note: *** F-statistics is significant at 1% level.

**Table 4:** Two Stage probit Regression for the Determinants of Child Survival in Comoros.

	All Children		Children 0–59 Month		Children 0–12 Months	
Childalive	Coeff	Z stat	Coeff	Z stat	Coeff	Z stat
Maternal fertility	0.007	−0.41	0.115	4.62	1.213	10.11
Maternal education	0.008	0.31	0.054	1.21	0.902	7.47
**Regions**						
Ndzouani region	−0.020	−0.30	−0.117	−1.01	0.091	0.7
Ngazidja region	−0.173	−2.46	−0.267	−2.19	0.596	2.59
Children are twins	−0.441	−4.25	−0.856	−4.76	−3.326	−7.99
Gender of child	−0.044	−1.13	−0.085	−1.29	0.373	2.82
Urban residence	0.182	3.54	0.177	1.92	−0.078	−0.42
Muslim religion	0.376	2.10	0.181	0.57	−1.699	−3.07
Male household head	0.099	2.34	0.392	5.42	1.479	8.37
Age of head	−0.000	−0.25	−0.029	−9.65	−0.165	−10.91
Telephone access	0.119	1.44	0.191	1.38	0.733	3.03
Share toilet	0.015	0.29	0.144	1.65	0.643	3.76
Number of business trips	−0.005	−2.05	−0.004	−1.40	−0.009	−1.71
Health insurance access	−0.108	−1.05	−0.045	−0.27	0.484	1.63
Away more than one month	0.006	0.08	0.062	0.54	0.255	1.10
Mosquito nets ownership	−0.062	−1.25	−0.042	−0.47	0.525	3.08
Not smoking tobacco	0.039	0.98	0.111	1.63	−0.155	−0.94
Number of unions	−0.087	−2.71	−0.218	−4.18	−0.309	−2.88
Breastfed immediately	0.331	6.02	0.858	9.97	2.846	11.54
Age at first birth	0.012	2.05	0.059	6.76	0.298	10.43
Wealth index	0.00	1.24	0.00	0.76	0.000	−0.71
Radio	−0.019	−0.43	−0.046	−0.61	−0.539	−3.73
Newspapers	−0.032	−0.21	0.089	0.36	−3.283	−5.68
Television	0.048	1.01	0.003	0.04	0.626	4.13
Education residual	−0.002	−0.3	0.005	0.12	−0.649	−6.04
Fertility residual	−0.075	−4.5	−0.876	−21.42	−3.532	−11.32
Constant	1.077	4.01	0.180	0.41	−10.26	−8.37
Number of observations	11,497		3605		1198	
LR Chi Square (22)	247.66 ***		1388.62 ***		1037.21 ***	

Note: *** Implies F-statistics is statistically significant at 1% level.
